# Vascular Effects of Low-Dose ACE2 Inhibitor MLN-4760—Benefit or Detriment in Essential Hypertension?

**DOI:** 10.3390/biomedicines10010038

**Published:** 2021-12-24

**Authors:** Andrea Berenyiova, Iveta Bernatova, Anna Zemancikova, Magdalena Drobna, Martina Cebova, Samuel Golas, Peter Balis, Silvia Liskova, Zuzana Valaskova, Katarina Krskova, Stefan Zorad, Ezgi Dayar, Sona Cacanyiova

**Affiliations:** 1 Centre of Experimental Medicine of Slovak Academy of Sciences, Institute of Normal and Pathological Physiology, 841 04 Bratislava, Slovakia; andrea.berenyiova@savba.sk (A.B.); Iveta.Bernatova@savba.sk (I.B.); anna.zemancikova@savba.sk (A.Z.); magdalena.drobna@savba.sk (M.D.); martina.cebova@savba.sk (M.C.); samuel.golas@savba.sk (S.G.); Peter.Balis@savba.sk (P.B.); Silvia.Liskova@savba.sk (S.L.); zuzana.valaskova@savba.sk (Z.V.); ezgi.dayar@savba.sk (E.D.); 2 Faculty of Medicine, Institute of Pharmacology and Clinical Pharmacology, Comenius University, 813 72 Bratislava, Slovakia; 3 Faculty of Medicine, Institute of Histology and Embryology, Comenius University, 811 04 Bratislava, Slovakia; 4 Biomedical Research Center Slovak Academy of Sciences, Institute of Experimental Endocrinology, 841 05 Bratislava, Slovakia; katarina.krskova@savba.sk (K.K.); stefan.zorad@savba.sk (S.Z.)

**Keywords:** essential hypertension, ACE2 inhibitor, SARS-CoV-2, Mas receptors, nitric oxide, hydrogen sulfide, angiogenesis, CAM

## Abstract

Severe acute respiratory syndrome coronavirus-2 (SARS-CoV-2) infects host cells through angiotensin-converting enzyme 2 (ACE2). Concurrently, the product of ACE2 action, angiotensin 1–7 (Ang 1–7), binds to Mas receptors within the cardiovascular system and provides protective effects. Therefore, it is crucial to reveal the role of ACE2 inhibition, especially within pre-existing cardiovascular pathologies. In our study, we imitated the action of SARS-CoV-2 in organisms using the low dose of the ACE2 inhibitor MLN-4760 with the aim of investigating to what degree ACE2 inhibition is detrimental to the cardiovascular system of spontaneously hypertensive rats (SHRs), which represent a model of human essential hypertension. Our study revealed the complex action of MLN-4760 in SHRs. On the one hand, we found that MLN-4760 had (1) (pro)obesogenic effects that negatively correlated with alternative renin-angiotensin system activity and Ang 1–7 in plasma, (2) negative effects on ACE1 inhibitor (captopril) action, (3) detrimental effects on the small arteries function and (4) anti-angiogenic effect in the model of chick chorioallantoic membrane. On the other hand, MLN-4760 induced compensatory mechanisms involving strengthened Mas receptor-, nitric oxide- and hydrogen sulfide-mediated signal transduction in the aorta, which was associated with unchanged blood pressure, suggesting beneficial action of MLN-4760 when administered at a low dose.

## 1. Introduction

Coronavirus-2 (SARS-CoV-2)-induced COVID-19 has been declared a global pandemic. Although the disease is manifested mainly by respiratory symptoms, in some patients it is associated with serious damage in the cardiovascular system. In addition, some pre-existing cardiovascular pathologies, such as hypertension, coronary heart diseases and diabetes, are risk factors that predispose patients with SARS-CoV-2 infection to serious complications or even death [1]. SARS-CoV-2 viral infection enters cells in the host via angiotensin-converting enzyme 2 (ACE2), a receptor for the spike protein of SARS-CoV-2 [2]. Walls et al. [3] not only showed that SARS-CoV-2 uses ACE2 to enter cells but also found that the SARSCoV-2 S glycoprotein harbors a furin cleavage site at the boun-dary between the S1/S2 subunits, which is processed during biogenesis and sets this virus apart from SARS-CoV-2 and SARS-related CoVs. This alters downstream signaling of angiotensin 1–7 (Ang 1–7), the main product of ACE2. In uninfected cells, Ang 1–7 binds to Mas receptors (proto-oncogene, G protein-coupled receptor) within the cardiovascular system and induces essential protective effects through its anti-inflammatory, antifibrotic and antioxidant action. In addition, activation of Mas receptors leads to the release of nitric oxide (NO), a well-known vasodilator [4,5]. Generally, ACE2-mediated mechanisms participate in the maintenance of normal endothelium-dependent relaxation, and endothelial dysfunction is reversed in ACE2-knockout mice [6] to promote endothelial dysfunction and inflammation [7]. Since binding of the SARS-CoV-2 virus to transmembrane ACE2 could result in dysfunction of Mas receptor-mediated pathways in the vasculature, it is crucial to reveal the role of ACE2 inhibitor-treatment, especially within pre-existing cardiovascular pathologies.

In our study, we imitated the assumed inhibition of ACE2-mediated signaling by SARS-CoV-2 using a low dose of the ACE2 inhibitor MLN-4760 with the aim of investigating to what degree the inhibition of ACE2 is detrimental to the cardiovascular system of hypertensive subjects. As an animal model, we used spontaneously hypertensive rats (SHRs), which represent a model of human essential hypertension. SHRs are characte-rized by increased systolic blood pressure, mean arterial pressure, cardiac and vascular wall hypertrophy, elevated systemic resistance, endothelial dysfunction and altered arterial contractility [8,9,10,11]. Moreover, Kodavanti et al. [12] have shown that SHRs show abnormalities in the lungs, which include bleeding, inflammation as well as increased oxidative stress associated with excessive cytokine production. In this respect, this experimental model of hypertension meets the criteria of cardiovascular complications as well as the features of lung injury observed in patients with COVID-19 disease. Although there are studies investigating the effects of ACE2 inhibition after treatment with MLN-4760 in diabetic mice and rats with acute respiratory distress syndrome [13,14], there is no information about the effect of MLN-4760 on the cardiovascular system of SHRs.

Nitric oxide (NO) is a crucial molecule of the arterial wall with a significant vasorelaxant effect, and its release is also required to counterbalance vasoconstriction [15,16]. Several authors confirmed that under physiological conditions, the stimulation of Mas receptors with Ang 1–7 led to the activation of endothelial NO synthase and NO release in endothelial cells [17,18]. Raffai and Lombard [19] showed that acute administration of Ang 1–7 unexpectedly restored suppressed acetylcholine-induced vasorelaxation in the arteries of rats fed a high salt content. Similarly, Savoia et al. [20] demonstrated that Mas receptor activation may contribute to improved vascular function in SHRs, which was associated with increased NO bioavailability. These data suggest vasoprotective effects of Mas receptor activation in restoring impaired endothelial function during various pathological stages. In addition, NO and Ang II also play an important role in angiogenesis. Studies have shown that angiogenesis is attenuated when NO bioactivity is reduced [21,22]. On the other hand, the effect of Ang II on vascular growth depends on the me-chanism of Ang II action: pro-angiogenic activity is mediated mainly by stimulation of angiotensin II receptor 1, whereas angiotensin II receptor 2 inhibits angiogenesis [23].

An important system associated with the NO signaling pathway is the next gaseous transmitter hydrogen sulfide (H_2_S). There is a lack of information about the interaction between H_2_S and ACE2. Lin et al. [24] showed that H_2_S donor administration increased ACE2 expression and Ang (1–7) production in endothelial cells, which in turn weakened atherosclerosis in mice with partially ligated carotid artery. On the other hand, MLN-4760 eliminated the protective anti atherosclerotic effect of H_2_S. Experiments using SHRs proved that H_2_S can interfere with the etiopathogenesis of hypertension. Tain et al. [25] demonstrated that the administration of a H_2_S donor inhibited ACE expression in the kidneys of SHRs and prevented the development of hypertension. Our previous study confirmed that SHRs could be endowed with compensatory vasoactive mechanisms, including potentiated H_2_S vasorelaxation [26]. Moreover, the activation of H_2_S signaling could represent a supplementary mechanism in cases of endogenous NO deficiency [11]. However, the role of endogenous H_2_S in SHRs in the interaction with the ACE2 pathway in vasoactive responses has not yet been evaluated.

The aim of this study was to investigate the effects of long-term s.c. infusion of the low-dose ACE2 inhibitor, MLN-4760, on blood pressure, RAS, adiposity and vascular functions, i.e., namely on NO and H_2_S-mediated mechanisms in the arteries of various size in SHRs, as well as the effect on angiogenesis in the model of chick chorioallantoic membrane (CAM). We hypothesized that the ACE2 inhibitor MLN-4760 deteriorates NO- and H_2_S-mediated relaxations and increases vascular contractility, which may result in further exacerbation of pre-existing hypertension in SHRs. We also assumed the anti-angiogenic effect of MLN-4760.

## 2. Materials and Methods

### 2.1. Guide for the Use and Care of Laboratory Animals

The SHRs used in this study were received from the accredited breeding facility of the Center of Experimental Medicine, Slovak Academy of Sciences, Dobrá Voda, Slovak Republic. Rats were bred in accordance with the institutional guidelines of the Ethical Committee of the Centre of Experimental Medicine. Experiments were performed in accordance with the European, Directive 2010/63/EU and approved by the State Veterinary and Food Administration of the Slovak Republic (Permit Number: 2652/2021-220, 19 March 2021). The animals were housed in a 12 h light/12 h dark cycle at constant humidity (45–65%) and temperature (20–22 °C) and had free access to standard laboratory rat chow (Altromin 1324P, Lage, Germany) and drinking water ad libitum.

### 2.2. Experimental Model and Blood Pressure Determination

Sixteen- to eighteen-week-old male SHRs (*n* = 40) were used in this study. The rats were divided into two groups: the control (SHR; *n* = 20) and MLN-treated groups (SHR + MLN; *n* = 20).

The specific ACE2 inhibitor MLN-4760 (MLN) (MedChemExpress, Monmouth Junction, NJ, USA) was administered using Alzet^®^ mini-osmotic pumps, model 2002, with a pumping rate of 0.5 μL/h for 14 days (DurectTM, Cupertino, CA, USA) at a dose of 1 mg/kg/day dissolved in 10% dimethyl sulfoxide in isotonic saline (sodium chloride 0.9% Braun intravenous solution for infusion; 308 mOsm/L; 250 mL; B. Braun Melsungen AG, Germany). In controls, mini-osmotic pumps were filled with 10% DMSO in isotonic saline. All pumps were implanted subcutaneously on the dorsum of rats under isoflurane inhalation (2.5–3%) anesthesia in aseptic conditions. The cut was closed by three stitches by braided non-absorbable silk suture (SMI, St. Vith, Belgium). Basal and final body weights (BWs) were determined 1 day before minipump implantation and on the last day of treatment, respectively. Each group comprised two separate subgroups: one for realization of in vivo studies (integrated blood pressure response (*n* = 8)) and one (*n* = 12) for in vitro functional and biochemical studies.

Systolic blood pressure (SBP) was measured using tail-cuff plethysmography (MRBP, IITC Life Science Inc., LA, CA, USA) 3 days before minipump implantation (basal). Final blood pressure (end) was investigated on day 12 (in subgroups for in vivo studies) or day 13 (in subgroups for in vitro studies) of treatment. All rats were trained for the tail-cuff method of blood pressure determination for three consecutive days before determination of basal levels of SBP. Five measurements were performed in each rat, and BP was calculated as the average of the last four measurements.

### 2.3. In Vivo Study—Integrated Blood Pressure Response

The rats were anesthetized with isoflurane inhalation anesthesia (2.5%, O_2_-1.5 L/min), which was continually maintained during the experiment. A cannula was inserted into the right jugular vein and heparin sulfate (25 IU, 100 μL) was administered to prevent coagulation. Subsequently, the right carotid artery was prepared, and a pressure sensor connected to a pressure transducer was inserted (FOP-LS-PT9–10, FISO Technologies, Quebec City, QC, Canada). After stabilization of BP within 15–25 min, the compounds dissolved in 100 μL saline were prepared and injected into the jugular vein over a 10 s period as follows: noradrenaline (NA, 1 µg/kg; agonist of adrenergic receptors; Zentiva, Prague, Czech Republic), acetylcholine (Ach, 1 µg/kg; agonist of muscarinic receptors), captopril (10 mg/kg; angiotensin-converting enzyme inhibitor), bismuth(III) subsalicylate (BSC, 0.25 µg/kg; hydrogen sulfide scavenger), and N^G^-nitro-L-arginine methylester (L-NAME, 30 mg/kg; NO synthase inhibitor). The responses were expressed as the mean arterial BP (MAP). The changes in MAP induced by Ach and NA were evaluated as the difference between values of maximal response after addition of the compound (blood pressure increase or decrease) and basal MAP (immediately before the addition of the compound). The responses induced by captopril, BSC and L-NAME, which developed long-lasting responses, were evaluated 10 min after injection. The response to acetylcholine (1 µg/kg) was carried out again after a 15 min pretreatment with L-NAME (30 mg/kg). Stock solutions of all compounds were prepared only at the time of measurement and used within a few hours. The recorded analogue signal was digitalized, and DEWESoft 6.6.7 software (DEWETRON, Prague, Czech Republic) was used for data acquisition and further analysis.

### 2.4. In Vitro Studies

On day 14 of treatment, the rats were killed by decapitation after brief CO_2_ anesthesia. After decapitation, trunk blood was collected into heparinized tubes (140 UI/5 mL), and aliquots of plasma and tissue samples (heart, retroperitoneal fat, epididymal fat) were rapidly weighed, frozen in liquid nitrogen, and stored at −80 °C for further analyses. The thoracic aorta (TA), femoral artery (FA) and first branches of the superior mesenteric artery (MA) were used for functional study in vitro.

#### 2.4.1. Preparation of Blood Samples for Determination of Biochemical Parameters in Plasma

Trunk blood was collected into preprepared heparinized tubes (140 UI/5 mL) and then centrifuged (850× *g*, 10 min, 4 °C, Centrifuge 5430 R, Eppendorf, Hamburg, Germany). Plasma was stored at −80 °C until the analysis of biochemical parameters of experimental animals. Plasma samples were allowed to thaw to room temperature before the measurement. Subsequently, 100 μL of plasma was pipetted into the sample chamber via the sample port. Then, 430 μL of distilled water was added into the diluent chamber via the diluent port of the test-specific reagent disk. Using auxiliary reagent discs (Celercare, MNCHIP Technologies Co., Ltd., Tianjin, China), we performed analyses of glucose (GLU), triacylglyceride (TG), cholesterol (CHOL) and high-density lipoprotein (HDL-C) levels in the plasma samples by a biochemical analyzer (Celercare, MNCHIP Technologies Co., Ltd., Tianjin, China).

#### 2.4.2. Analysis of Angiotensins

The equilibrium levels of angiotensin (1–10) (Ang I), angiotensin (1–8) (Ang II), angiotensin (1–7) and angiotensin (1–5) peptide concentrations were determined by mass spectrometry LC-MS/MS in frozen plasma samples as described previously [27]. This ex vivo analysis determines the established equilibrium rate between angiotensin peptide’s production and elimination and takes all plasma RAS soluble factors of angiotensins production/elimination into account. The analysis thus makes it possible to calculate total alternative RAS activity (ALT-S), as a ratio of (Ang 1–7 + Ang 1–5)/(Ang I + Ang II + Ang 1–7 + Ang 1–5) as well as ACE activity as ratio of Ang II/Ang I. 

#### 2.4.3. Measurement of H_2_S Concentration

H_2_S concentration was measured in plasma and heart tissue via methylene blue as-say as described previously [28,29]. To assess H_2_S production, 75μL of plasma combined with 500 μL of reaction mixture containing 0.1 mol/L potassium phosphate buffer (325 μL), substrate, and cofactor of H_2_S that are l-cysteine and pyridoxal-5-phosphate, respectively. To measure H_2_S generation from the heart, tissue was homogenized with lysis buffer containing sodium orthovanadate and protease inhibitor. Protein concentration was determined by Lowry assay. Homogenates of samples (total concentration of protein was 50 µg) were added to a reaction mixture (total volume 500 µL) containing potassium phosphate buffer, pyridoxal-5-phosphate, and l-cysteine to measure H_2_S generation [30].

For both plasma and heart tissue, samples were incubated for 30 min at 37 °C. After the reaction, trichloroacetic acid (10%, 250 mL) was added to the mixture followed by zinc acetate (1%, 250 mL), N,N-dimethyl-p-phenylenediamine sulfate (20 mmol/L, 133 mL) in 7.2 mol/L HCl, and FeCl_3_ (30 mmol/L, 133 mL) in 1.2 mol/L HCl incubation to trap H_2_S and to precipitate proteins. After 10 minutes of incubation at room temperature, the mixtures were centrifugated for 5 min, 8944× *g*, 4 °C. The absorbance of resulting solution was measured at 650 nm with a spectrophotometer (NanoDrop™ 2000/2000c Spectrophotometers, Thermo Fisher Scientific, Waltham, MA USA). In a 96-well plate, all standards and samples were assayed in duplicated. The H_2_S concentration of each sample was calculated against a calibration curve of (Na_2_S; 3.9–250 mmol/L) and results show the plasma H_2_S concentration in μmol/L, and the heart results are expressed as nmol/mg of protein.

#### 2.4.4. Vasoactive Responses of Thoracic Aorta

The TA from the aortic arch to the diaphragm was isolated and the surrounded adipose and connective tissues were removed. The isolated TA then was cut into rings (5 mm in length) which were vertically fixed due to two stainless steel wire triangles established into the lumen of the TA. The rings were placed in a 20 mL organ bath with Krebs solution (oxygenated with 95% O_2_ and 5% CO_2_, 37 °C) containing 118 NaCl mmol/L, 5 KCl mmol/L, 25 mmol/L NaHCO_3_, 1.2 mmol/L MgSO_4_, 1.2 mmol/L KH_2_PO_4_, 2.5 mmol/L CaCl_2_, 11 mmol/L glucose, 0.032 mmol/L Ca-Na_2_EDTA. While the bottom triangle was immovably fixed, the upper wire triangle was connected to isometric tension sensors (FSG-01, MDE, Budapest, Hungary), and changes in tension were registered by an NI USB-6221 AD converter (MDE, Budapest, Hungary) and S.P.E.L. Advanced Kymograph software (MDE, Budapest, Hungary). To each TA ring a resting tension of 1 g was applied, and the TA ring were stabilized during a 45 to 60 min lasting equilibration period in order to avoid a nonspecific stress relaxation [31,32].

Ach (10^−5^ mol/L) was applied on the NA (10^−6^ mol/L, 10^−7^ mol/L)-precontracted arte-ries to test the integrity of the endothelium and the contractile abilities of the smooth muscle cells in TA rings, the concentration-dependent contractile responses were determined by exogenous NA applied as increasing doses (10^−10^–10^−5^ mol/L). The contractile responses were expressed as the developed tension in grams and normalized according to the length (mm) of the particular ring preparation. To express the sensitivity of the adrenergic receptors to NA the NA-induced contractions were expressed as percentages of the maximal reached contraction. From individual concentration-response curves concentrations producing the half-maximum of NA response (EC_50_) were assigned and expressed as the negative logarithm of NA molar concentration. The endothelium-dependent vasorelaxation was determined on the NA- precontracted (10^−6^ mol/L) TA rings. After reaching the steady state concentrations of Ach were applied cumulatively (10^−10^–10^−5^ mol/L). The rate of relaxation was calculated as a percentage of the contraction induced by NA.

A-799 trifluoroacetate salt (10^−5^ mol/L, 20 min), a selective Mas receptor inhibitor, was used to determine the participation of Mas receptors in vasoactive responses. TA rings were incubated with a nonspecific inhibitor of NO synthase, L-NAME (10^−5^ mol/L, 20 min), to evaluate the vasoactive effect of endogenous NO. To evaluate the participation of endogenous H_2_S in the vasoactive responses, the rings of TA were incubated with a H_2_S scavenger, BSC (10^−5^ mol/L, 20 min). All drugs were acutely incubated for 20 min in the organ bath to follow their effect on adrenergic contraction (NA, 10^−6^ mol/L or 10^−7^ mol/L) and endothelium-derived relaxation (Ach, 10^−10^–10^−5^ mol/L).

#### 2.4.5. Vasoactive Responses of Femoral and Mesenteric Arteries

The femoral artery (FA) was carefully isolated, and the connective tissues were removed, the FA were then immersed to the modified physiological salt solution (PSS: 118.99 mmol/L NaCl, 4.69 mmol/L KCl, 25 mmol/L NaHCO_3_, 1.17 mmol/L MgSO_4_.7H_2_O, 1.18 mmol/L KH_2_PO_4_, 2.5 mmol/L CaCl_2_.2H_2_O, 0.03 mmol/L Na_2_EDTA, 5.5 mmol/L glucose, pH 7.4) oxygenized with 95% O_2_ and 5% CO_2_. After that, the segments of FA were placed in a small vessel wire myograph chamber (Dual Wire Myograph System 510A, DMT A/S, Aarhus, Denmark). Similarly, the mesenteric arterial bed (intact with connected intestinal system) was carefully dissected and transferred to cold PSS, and the first branches of the superior mesenteric artery (MA) were cleaned of adipose and connective tissues. The rings of MA were placed in a wire myograph chamber for small vessels (Dual Wire Myograph System 620M, DMT A/S, Aarhus, Denmark). In both, the femoral and mesenteric arterial segments, the inner arterial diameter was adjusted to 90% of the diameter predicted for the pressure of 100 mmHg in the myograph chamber. Then, the arteries were stabilized for 30 min to achieve their basal tones. The arteries were incubated for 2 min in a depolarizing solution (125 mmol/L K^+^, NaCl was exchanged for an equimolar concentration of KCl) to examine their viability as described by Balis et al. 2020 [33].

(A) Recording of the vascular reactivity of the femoral artery

After a 30 min stabilization period, arterial contractions of the FA were induced by cumulative doses of NA (10^−8^–10^−4^ mol/L). Then, Ach-induced relaxations were measured after cumulative application of Ach (10^−9^–10^−5^ mol/L). Concentration-response curves were constructed, and maximal efficacy and half maximal effective concentration were assessed using the Hills equation as described previously [34,35].

(B) Recording of the vascular reactivity of the mesenteric artery

The MA segments were pre-constricted with NA (10^−6^ mol/L). After reaching a steady state of NA contraction, increasing cumulative concentrations of Ach (10^−9^–10^−5^ mol/L) were added to record endothelium-dependent relaxation.

#### 2.4.6. Total NO Synthase Activity

Total NOS activity was determined in the 10% of aorta homogenates by measuring [3H]-L-citrulline formation from [3H]-L-arginine (MP Biochemicals, Santa Ana, California, USA) using the Quanta Smart Tri-Carb Liquid Scintillation Analyzer (TriCarb, Packard, UK) as described elsewhere [36]. The results are expressed as picokatal per gram of protein (pkat/g protein).

#### 2.4.7. RNA Isolation and Real-Time PCR

RNA was segregated from deep-frozen abdominal aorta tissue samples (−80 °C) by application of Minilys personal homogenizer (Bertin Technologies SAS, Montigny-le-Bretonneux, France) and RNeasy Fibrous Tissue Mini Kit (Qiagen, Valencia, CA, USA) following the manufacturer´s directions. Reverse transcription of isolated RNA was achieved by High-Capacity cDNA Reverse Transcription Kit (Thermo Fisher Scientific, Waltham, MA, USA) in accordance with the manufacturer’s protocol. Real-time qPCR took place in FastStart Universal SYBR Green Master mix solution (Roche, Indianapolis, Indiana) and QuantStudio™ 5 Real-Time PCR System (Applied Biosystems, Thermo Fisher Scientific, Waltham, MA, USA) using rat-specific primer pairs for Ace2, Fw: 5′-TCAGAGCTGGGATGCAGAAA-3′ and Rv: 5′-GGCTCAGTCAGCATGGAGTTT-3′; Mas1, Fw: 5′-TGACCATTGAACAGATTGCCA-3′ and Rv: 5′-TGTAGTTTGTGACGGCTGGTG-3′. Data were normalized to the expression of the housekeeping gene TATA box binding protein (Tbp), which was not altered by the treatment.

#### 2.4.8. Western Blotting

Protein expression levels of all NOS isoforms, CSE, CBS, ACE2 and the Mas receptor, were determined in the aorta by Western blot analysis. Briefly, aortic samples were homogenized in cold lysis buffer containing 0.05 mmol/L Tris and protease inhibitor cocktail and centrifuged at 15,000 rpm at 4 °C for 20 min. Protein concentrations of supernatant were determined by Lowry assay. Equal amounts of samples were subjected to SDS-PAGE using 12% gels and transferred to nitrocellulose membranes at room temperatures. Membranes were blocked with 5% nonfat milk for 1 h at room temperature in Tris-buffer solution (pH 7.6) containing 0.1% Tween-20 and probed against the following primary antibodies: rabbit polyclonal anti-endothelial NOS, anti-neuronal NOS and anti-β-actin (Abcam, Cambridge, UK); rabbit polyclonal anti-inducible NOS (Bio-Rad, Inc., Hercules, CA, USA) rabbit polyclonal anti-angiotensin(1–7) Mas receptor (Alomone Labs, Jerusalem, Israel); rabbit polyclonal CBS and mouse monoclonal anti-CSE antibodies (Proteintech, Manchester, UK); and rabbit monoclonal anti-ACE2 (Invitrogen, Waltham, MA, USA) overnight at 4 °C. Antibodies were detected using a secondary horseradish peroxidase-conjugated anti-rabbit antibody (Abcam, Cambridge, UK) or anti-mouse antibody (Cell Signaling Technology, Danvers, MA, USA) at room temperature for 2 h. The immunoreactive bands were visualized using an enhanced chemiluminescence system (ECL, Amersham, UK) and quantified by using a Chemi-DocTM Touch Imaging System (Image LabTM Touch software, BioRad, Inc., Hercules, CA, USA). β-actin was used as an internal loading control.

#### 2.4.9. Measurement of ACE2 Activity

The activity of ACE2 was determined in abdominal aorta lysates using a fluorometric assay kit (Angiotensin II Converting Enzyme Activity Assay Kit, BioVision, Inc., Milpitas, CA, USA). Frozen aortae were homogenized using a glass Teflon homogenizer in lysis buffer in accordance with the manufacturer’s instructions, and lysates obtained by centrifugation at 16,000× *g*/10 min/4 °C were used to measure the protein concentration (bicinchoninic acid protein assay; Sigma-Aldrich, St. Louis, MO, USA) and ACE2 activity. The assay procedure utilizes the ability of active ACE2 to cleave a synthetic MCA-based peptide substrate to release free MCA (7-methoxycoumarin-4-acetate), quantified using a fluorescence microplate reader (Synergy™ H4 Hybrid Reader; BioTek, Winooski, VT, USA) and enzyme kinetic measurements over 120 min at room temperature with data collection in 5-minute intervals at wavelengths Ex/Em = 320/420 nm. Enzyme kinetics were measured in the absence (negative control) or presence of an ACE2-specific inhibitor to distinguish ACE2 activity from other proteolytic activities.

### 2.5. Evaluation of Angiogenesis in the Model of Chick Chorioallantoic Membrane

To evaluate the effect of MLN-4760 on angiogenesis, an ex ovo model of the chick chorioallantoic membrane (CAM) was used as described previously [37,38], with some modifications. Fertilized white Leghorn chicken eggs were purchased from a local producer (Liaharenský podnik a.s. Nitra, Slovakia). The eggs were placed in a vertical position, with the round tip up in an automatic egg incubator at 37.8 °C, 65–70% humidity and rotation angle of rotation 35°, every 120 min) for 96 h. On the fifth day of embryonic development (D5), the egg shell was sawn with a laboratory wheel saw, and the egg content was gently placed in a sterilized polypropylene medical cup. CaCO_3_ was added (10 mg, outside the yolk area), and the cups were covered with food foil using a sterilized rubber band. The cups were placed in an incubator (Memmert IF160, Schwabach, Germany) at 37.5 °C and 60–65% humidity for 72 h. On D8, polytetrafluoroethylene (Teflon) circles were placed on the surface of developing CAM. On the underside of the circles, 0.45% methylcellulose gel containing 10% DMSO (control) or 5 µg MLN-4760 dissolved in 10% DMSO was applied (*n* = 16 in each group). After 72 h of incubation, CAM status was examined with a Motic SMZ171 trinocular stereo microscope (Motic, Kowloon, Hong Kong) equipped with a Bresser MikroCamII microscope 12 MP USB 3.0 camera (Bresser, Rhede, Germany). Areas of the circles were photographed after gentle removal of the Teflon circle. The pictures were then evaluated using MicroCamLabII (Bresser, Rhede, Germany) software. To improve the image, the colors were inverted, and the contrast was increased to achieve the best possible resolution. All vascular branching points (bifurcations) were counted in the area of contact of the applied substance (A_Appl_) with a diameter of ~6.2 mm and in the area adjacent to A_Appl_ (A_Adj_, diameter ~9.3 mm, i.e., plus 50% of A_Appl_ diameter), and the number of branchings per mm^2^ was counted in each area. Each CAM was examined by two evaluators, and the average values of their evaluations were statistically evaluated (*n* = 8 per group). At the end of the experiment, the embryos were killed by decapitation. The number of preliminarily dead embryos was similar in both groups (50%). A detailed description of the method of preparation of the ex ovo model of chick CAM and Teflon disc preparation is described in Supplementary File S1.

### 2.6. Statistical Analysis

The data were expressed as the mean ± S.E.M. Two-way and three-way analysis of variance (ANOVA) for repeated measurements with the Bonferroni post hoc test were used to evaluate of vasoactive responses. To compare cardiovascular and plasmatic data, NO synthase and ACE2 activities, H_2_S concentrations, gene and protein expression, and the vasoactive response to a single dose of compound, Student’s *t*-test was used. The effect of MLN-4760 on angiogenesis was analyzed by 2-way ANOVA (group × area). Correlations between variables were evaluated using Pearson’s correlation coefficient (r). The differences between the means were assessed as significant at *p* < 0.05. Data were analyzed using OriginPro (OriginLab Corporation, Northampton, MA, USA), GraphPad Prism 7.0 (GraphPad Software, Inc., La Jolla, CA, USA) and Statistica 13.5 (StatSoft, Hamburg, Germany).

### 2.7. Drugs

All the chemicals used in this study were purchased from Merck (Bratislava, Slovakia) unless stated otherwise.

## 3. Results

### 3.1. General Characteristics of Experimental Animals

The end SBP and relative increment of SBP (Δ BP%) as well as the heart weight and heart weight to body weight ratio were unchanged in SHRs treated with MLN-4760 compared to control rats. Although there were no changes in the values of end body weight (BW), ACE2 inhibition by MLN-4760 resulted in a significant increase in the relative body weight increment (Δ BW (%), Table 1). An important contributor to the above increase in body weight seems to be visceral adipose tissue (expressed as a sum of retroperitoneal and epididymal fat weights), which itself shows a significant mass elevation as well as when expressed as the adiposity index (Table 1). The ACE2 activity in TA tissue was comparable between the groups (Table 1). The levels of H_2_S in plasma and the heart were significantly increased in SHRs treated with MLN-4760 compared to control rats (Table 1). The effect of ACE2 inhibition on glucose metabolism was represented by a strong tendency toward elevated blood glucose in the MLN-4760 group (Table 1, *p* = 0.0596). The treatment had no effect on the plasma levels of TG, CHOL and HDL-C (Table 1).

### 3.2. Analysis of Angiotensins in Plasma

**Figure 1:**
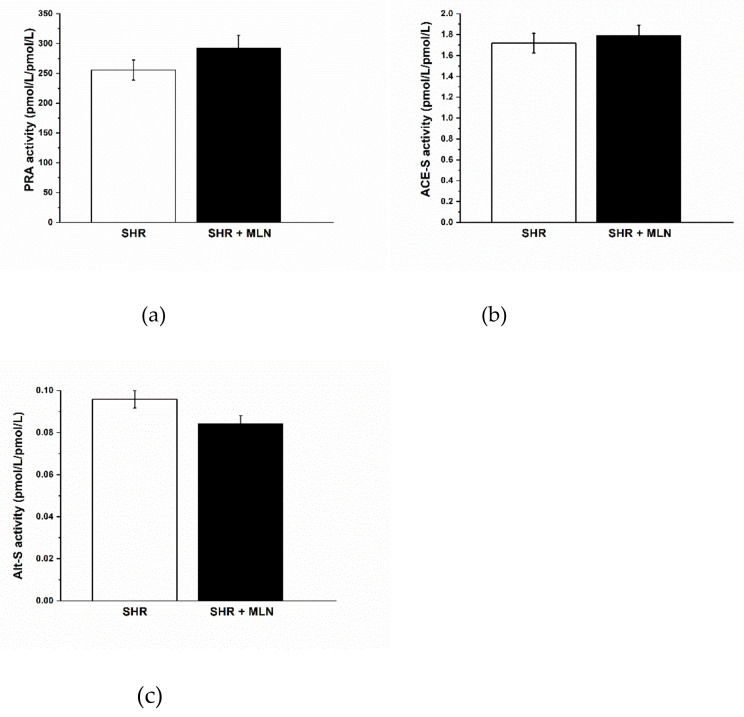
The effect of MLN-4760 treatment on the activity of soluble RAS enzymes determined from angiotensin product/substrate ratios in plasma–plasma renin activity, PRA (**a**); soluble angiotensin converting enzyme, ACE-S (**b**); and the total alternative renin angiotensin system activity, Alt-S (**c**).

**Figure 2:**
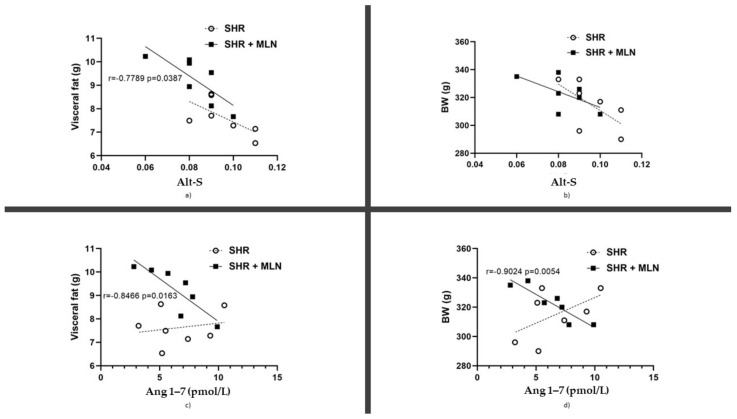
The correlation between the plasma level of the total alternative renin angiotensin system activity (Alt-S) and the weight of the visceral fat (**a**) and body weight (**b**). The correlation between the plasma level of angiotensin 1–7 (Ang 1–7) and the weight of the visceral fat (**c**) and body weight (**d**) in SHRs and SHRs treated with MLN-4760 (SHR + MLN).

Treatment with MLN-4760 did not alter the plasma levels of Ang I, Ang II, Ang 1–7, Ang 1–5 or Ang IV (Table 2). Similarly, it had no impact on plasma renin activity (PRA, Figure 1a) or ACE-S activity (Figure 1b); however, it had a tendency to reduce total alternative RAS activity (Alt-S) (*p* = 0.061, Figure 1c). The importance of alternative arms of the RAS in the regulation of body weight through at least visceral fat is documented by correlations between Alt-S activity and Ang 1–7 concentration and BW and visceral fat mass (Figure 2). Interestingly, the negative correlation between Alt-S and Ang 1–7 and BW as well as visceral fat mass was statistically significant only in the MLN-4760-treated group.

### 3.3. In Vivo Vasoactive Responses

**Figure 3:**
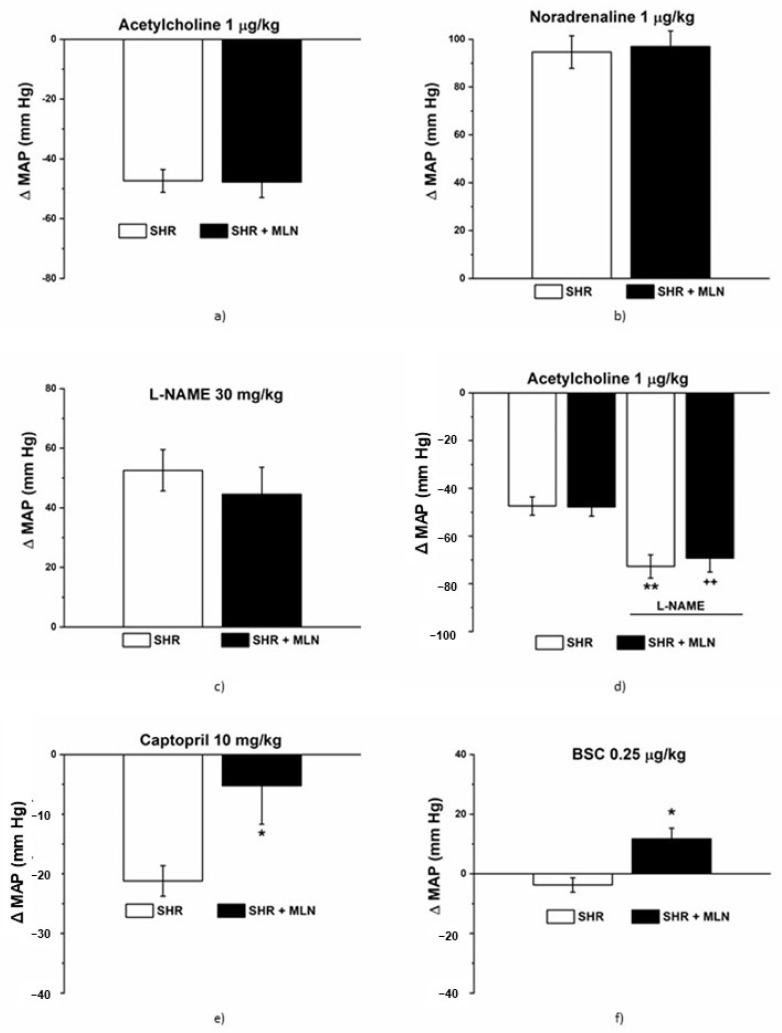
The effect of MLN-4760 treatment on the integrated pressure responses of the cardiovascular system. The changes in mean blood pressure (ΔMAP) were followed after infusion of acetylcholine (1 µg/kg, (**a**)), noradrenaline (1 µg/kg, (**b**)), NOS inhibitor (L-NAME, 30 mg/kg, (**c**)), ACE inhibitor-captopril (10 mg/kg, (**e**)) and H_2_S scavenger bismuth(III) subsalicylate (BSC, 0.25 µg/kg, (**f**)). The effect of acetylcholine (1 µg/kg) also was followed before and after pretreatment with L-NAME (30 mg/kg, (**d**)). The results are presented as the mean ± S.E.M., and differences between groups were analyzed by Student´s *t*-test. * *p* < 0.05, ** *p* < 0.01 vs. SHR; ^++^ *p* < 0.01 vs. SHR + MLN.

At the beginning of the experiment, the baseline values of MAP were comparable in the control (96.30 ± 5.4 mmHg, *n* = 8) and MLN-4760-treated rats (108.90 ± 4.01 mmHg, *n* = 8). Noradrenaline (NA, 1 μg/kg) induced a similar hypertensive response in rats treated with MLN-4760 (*n* = 8) compared to the response in the control group (*n* = 8), and the increase in MAP was comparable between the two groups (Figure 3a). Acetylcholine (Ach, 1 μg/kg) induced a similar hypotensive response in rats treated with MLN-4760 (*n* = 7) compared to the response in the control group (*n* = 6), and the decrease in MAP was comparable between the two groups (Figure 3b). N^G^-nitro-L-arginine-methyl ester (L-NAME, 30 mg/kg) induced a similar hypertensive response in rats treated with MLN-4760 (*n* = 8) compared to the response in the control group (*n* = 8), and the increase in MAP was comparable between the two groups (Figure 3c). Acute pretreatment with L-NAME significantly increased the hypotensive response induced by Ach in both the control (*n* = 8) and MLN-4760-treated groups (*n* = 7), and the decrease in MAP induced by Ach was comparable in both groups (*p* < 0.05, Figure 3d). Captopril (10 mg/kg) induced a hypotensive response in the control (*n* = 7) and MLN-4760-treated groups (*n* = 8); however, the decrease in MAP was significantly lower in the MLN-treated group (*p* < 0.05, Figure 3e). Bismuth(III) subsalicylate (BSC, 0.25 µg/kg) induced a mild hypotensive response in the control group (*n* = 8); on the other hand, its addition led to an increase in MAP in the MLN-treated group (*n* = 8, *p* < 0.05, Figure 3f).

### 3.4. Endothelial Function and Contractile Properties of Isolated Arteries

**Figure 4:**
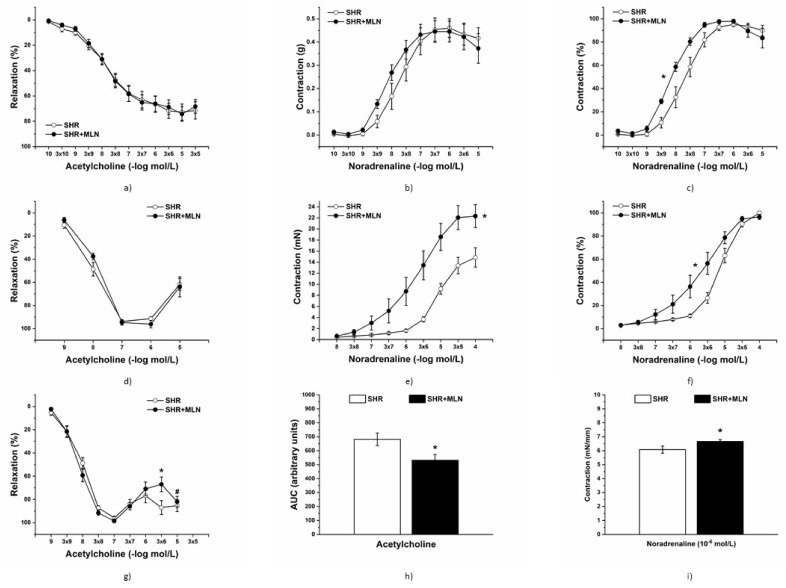
Endothelium-dependent vasorelaxant response of the thoracic aorta (**a**), femoral artery (**d**) and mesenteric artery ((**g**,**h**)-AUC (area under curve) values). Noradrenaline-induced contractile response of the thoracic aorta ((**b**)-absolute response, (**c**)-percent values of the maximum response), femoral artery ((**e**)-absolute response, (**f**)-percent values of the maximum response) and mesenteric artery ((**i**)-absolute response). Arteries were isolated from control SHRs and SHRs treated with MLN-4760. The results are presented as the mean ± S.E.M., and differences between groups were analyzed by repeated two-way repeated measures ANOVA or Student´s *t*-test, as appropriate. * *p* < 0.05 vs. SHR; ^#^ *p* < 0.05 vs. maximal response to Ach.

Treatment with MLN-4760 did not alter the endothelium-derived relaxation of TA rings (*n* = 8 in the control group, *n* = 8 in the MLN group, Figure 4a). Similarly, the maximum force of the adrenergic contraction was not changed after MLN-4760 treatment; however, it shifted the concentration-response curve to exogenous noradrenaline to the left (Figure 4b,c). Two-way ANOVA revealed a significant effect of MLN-4760 treatment on the sensitivity of the adrenergic receptors (F_(1;125)_ = 19.33, *p* = 2.64 × 10^−5^), and the EC_50_ values also were increased in the MLN-4760-treated group compared to the control SHRs (8.34 ± 0.1 mol/L vs. 7.83 ± 0.14 mol/L, *p* < 0.05). Treatment with MLN-4760 did not alter the endothelium-derived relaxation of FA rings (*n* = 7 in the control group, *n* = 8 in the MLN group, Figure 4d).The maximal NA-induced contraction of FA isolated from MLN-4760-treated animals (*n* = 8) was increased (MLN-4760: 23.51 ± 2.19 vs. SHR: 15.55 ± 1.71 mN, F_(8, 216)_ = 4.9949, *p* = 0.00001), and the sensitivity was shifted to the left, thus increasing as well (EC_50_ MLN-4760: 6.15 ± 0.23 vs. SHR: 5.36 ± 0.12 mol/L, *p* < 0.05) when compared to the control SHRs (*n* = 7, Figure 4e,f). Although there was no difference in the concentration-response curve to Ach between the two experimental groups in isolated MA, we observed reduced endothelium-dependent relaxation at the 3x10^−6^ mol/L Ach concentration, and the measured area under the curve (AUC, *p* < 0.05) was reduced in the MLN-4760-treated group (Figure 4g,h). At the same time, we observed a reduced relaxation capacity of MA compared to maximal Ach-induced relaxation (*p* < 0.05, Figure 4g), which indicates an increased production of endothelium-dependent vasoconstricting factor. In the MLN-4760 group, we observed increased NA-induced contraction of MA (10^−6^ mol/L, Figure 4i).

#### 3.4.1. Analyses of Mas Receptor Pathway

**Figure 5:**
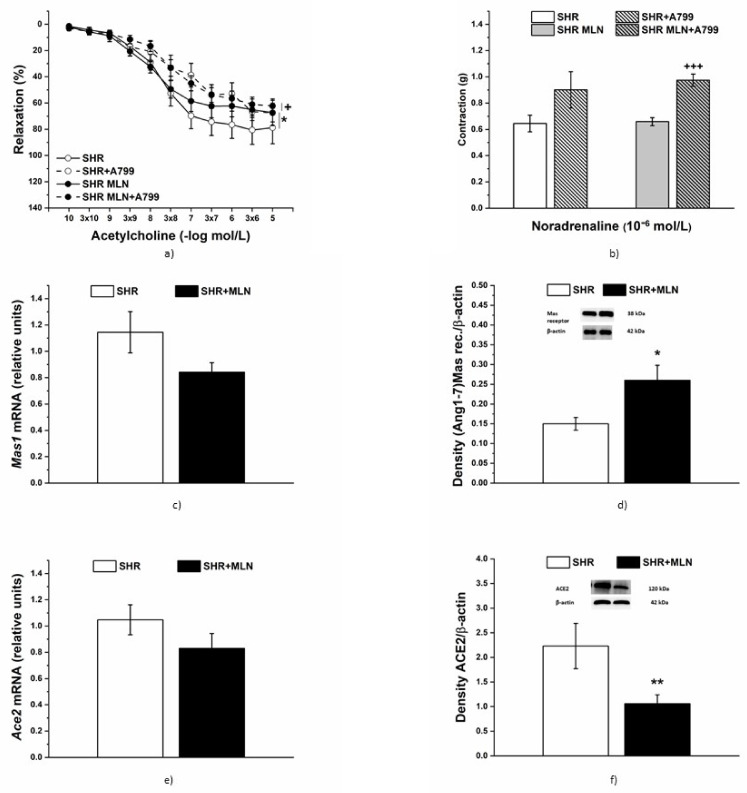
The effect of acute Mas receptor inhibition on the endothelium-dependent vasorelaxation (**a**) and contractile response (**b**) of TA isolated from control SHRs and SHRs treated with MLN-4760. The mRNA levels of the *Mas1* and *ACE2* genes (**c**,**e**) and the protein expression of the Mas receptor and ACE2 (**d**,**f**) were measured in aortic tissue. The results are presented as the mean ± S.E.M., and differences between groups were analyzed by three-way ANOVA or Student’s *t*-test, as appropriate. * *p* < 0.05 vs. SHRs, ** *p* < 0.01 vs. SHRs; ^+^ *p* < 0.05, ^+++^ *p* < 0.001 vs. SHR + MLN.

The acute inhibition of Mas receptors reduced the vasorelaxation of TA in both control SHRs (*n* = 8) and MLN-4760-treated SHRs (*n* = 8, Figure 5a). There was a significant effect of the A-799 trifluoroacetate salt (10^−5^ mol/L) on the relaxation response to Ach (F_(1;317)_ = 17.22, *p* = 4.44 × 10^−5^). On the other hand, pretreatment with A-799 trifluoroacetate salt significantly increased the contractile response to NA, which was observed only in SHRs treated with MLN-4760 (*p* < 0.001, Figure 5b). There was no difference between the control and MLN-treated groups in Ace2 mRNA levels (Figure 5e); however, MLN-4760 treatment tended to reduce the mRNA level of Mas receptor (*p* = 0.084, Figure 5c). The Mas receptor protein expression level was significantly increased in the aorta of the SHR-treated group compared to that of untreated rats (*p* < 0.01, Figure 5d). The opposite effect was found for ACE2 protein expression, which was decreased after MLN-4760 administration (*p* < 0.01, Figure 5f).

#### 3.4.2. Analyses of NO Pathway

**Figure 6:**
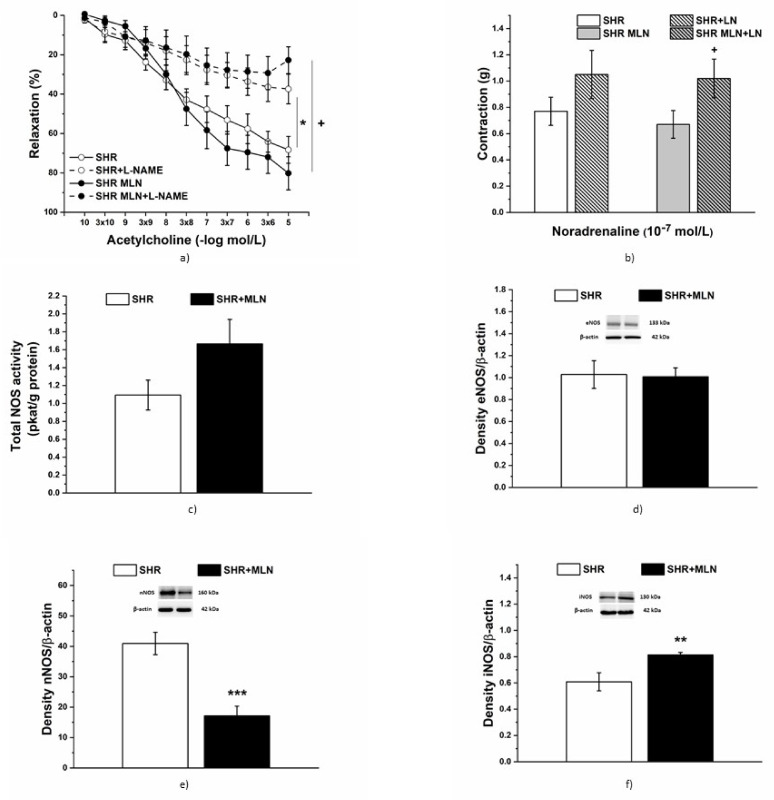
The effect of acute NO synthase (NOS) inhibition on endothelium-dependent vasorelaxation (**a**) and the contractile response (**b**) of TA isolated from control SHRs and SHRs treated with MLN-4760. The total NOS activity (**c**) and the protein expression of endothelial eNOS (**d**), neuronal nNOS (**e**) and inducible iNOS (**f**) were measured in aortic tissue. The results are presented as the mean ± S.E.M., and differences between groups were analyzed by three-way ANOVA or Student´s *t*-test, as appropriate, * *p* < 0.05, ** *p* < 0.01 vs. SHR, *** *p* < 0.001 vs. SHR; ^+^ *p* < 0.05 vs. SHR + MLN.

NO synthase inhibition by L-NAME (10^−5^ mol/L) significantly inhibited the endothelium-derived relaxation of TA, similarly in control SHRs (*n* = 8) and MLN-4760-treated rats (*n* = 8, Figure 6a). Three-way ANOVA revealed a significant effect of the L-NAME and Ach-concentration interaction on the relaxant response (F_(1;352)_ = 5.65, *p* = 2.76 × 10^−8^). However, treatment with L-NAME increased NA-induced contraction only in SHRs treated with MLN-4760 (*p* < 0.05, Figure 6b). Total NO synthase (NOS) activity of the aorta after MLN-4760 treatment tended to be significantly increased in comparison to that of the control SHR group (*n* = 12, *p* < 0.0779, Figure 6c). The endothelial NOS (eNOS) expression level was not changed after MLN-4760 treatment in comparison to the SHR group (*n* = 10; Figure 6d). A significantly decreased level of neuronal NOS (nNOS) was found in the MLN-4760-treated group (*n* = 10; *p* < 0.05, Figure 6e). On the other hand, we found upregulation of inducible NOS (iNOS) in this group (*n* = 10; *p* < 0.01, Figure 6f).

#### 3.4.3. Analyses of H_2_S Pathway

**Figure 7:**
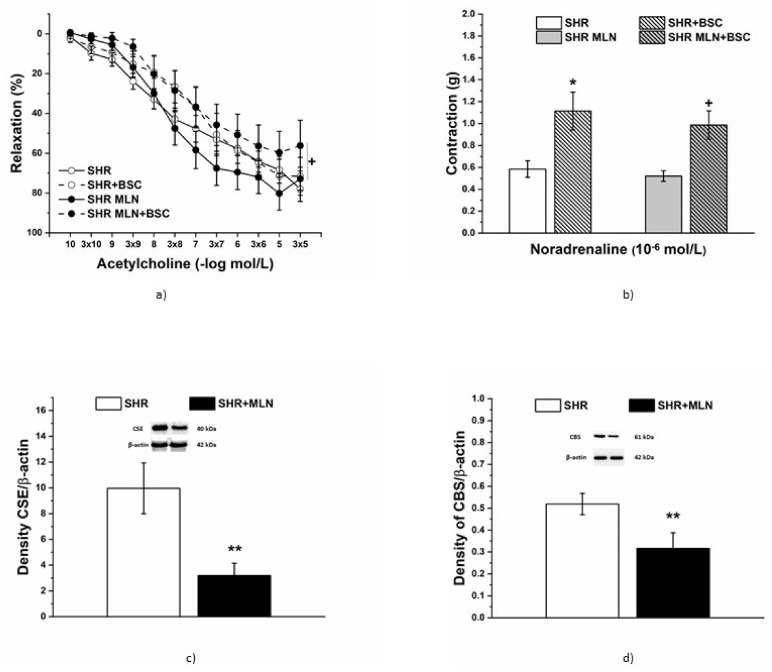
The effect of the H_2_S scavenger on endothelium-dependent vasorelaxation (**a**) and the contractile response (**b**) of TA isolated from control SHRs and SHRs treated with MLN-4760. The protein expression of cystathionine γ-lyase CSE (**c**) and cystathionine-β-synthase CBS (**d**) was measured in aortic tissue. The results are presented as the mean ± S.E.M., and differences between groups were analyzed by three-way ANOVA or Student´s *t*-test, as appropriate. * *p* < 0.05 vs. SHR, ** *p* < 0.01 vs. SHR; ^+^ *p* < 0.05 vs. SHR + MLN.

Incubation with BSC (10^−5^ mol/L), a H_2_S scavenger, significantly reduced endothelium-dependent vasorelaxation in the aorta only in SHRs treated with MLN-4760 (*n* = 8); moreover, preincubation with BSC itself (F_(1;326)_ = 17.53; *p* = 3.79 × 10^−5^) and treatment × incubation interaction had a significant effect on this response (F_(1;326)_ = 4.29; *p* = 0.039, Figure 7a). Scavenger application increased the adrenergic contractile response in both experimental groups (*n* = 8 in the control group, *p* < 0.05, *n* = 8 in the treated group, *p* < 0.05 Figure 7b). Similarly, both CSE and CBS protein expression levels in the aorta were significantly decreased (*p* < 0.01) in the MLN-4760 group compared to the SHR group (Figure 7c,d).

### 3.5. Effect of MLN-4760 on Angiogenesis in Chick CAM Model

**Figure 8:**
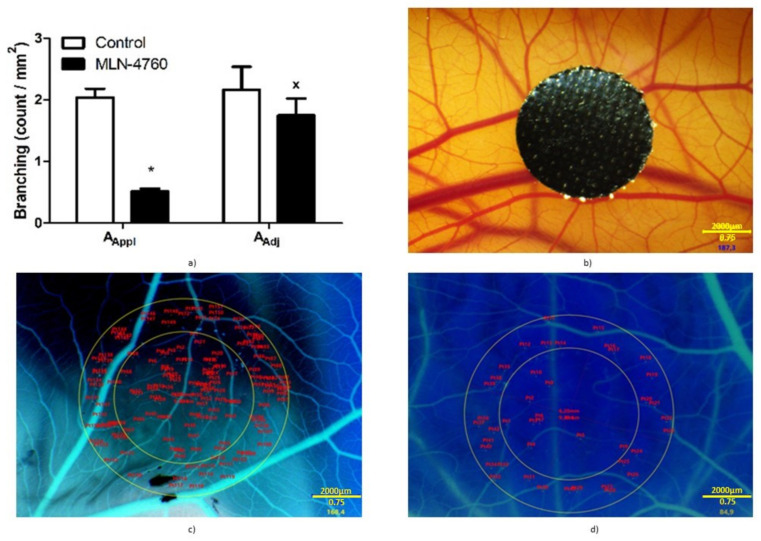
MLN-4760 reduces vascularization in the model of chick chorionallantoic membrane, determined as the number of vascular branching points in the area of MLN-4760 application compared to the area adjacent to the area of application (**a**). * *p* < 0.02 vs. SHR, ^x^ *p* < 0.02 vs. A_Appl_ in the given group. Results are analyzed by two-way ANOVA. Next pictures are examples of chick chorionallantoic membrane in native yellow color with black Teflon circle placed on chick chorionallantoic membrane (**b**), and examples of evaluations of vascular branching in pictures of chick chorioallantoic membrane with colors inverted to blue in the control group (**c**) and in MLN-4760-treated chorioallantoic membranes (**d**) after the removal of Teflon circles. Abbreviations: A_Appl_, area of drug application; A_Adj_, area adjacent to area of drug application.

MLN-4760 significantly reduced the number of vascular branches (bifurcations) in the area of application by ~75% (*p* < 0.01) compared to the control group (Figure 8a). The number of vascular branches in the A_Adj_ significantly increased in the MLN-4760-treated CAM, which was not found in the control group. In addition, hypervascularization was clearly visible in A_Appl_ in MLN-4760-treated CAMs. Examples of native CAM and CAM with inverted colors for easier determination of vascular branches are shown in Figure 8b–d.

## 4. Discussion

In this study, we intended to develop a noninfectious pharmacological model of cardiovascular complications associated with COVID-19 due to altered ACE2 function. We found that, despite the treatment of spontaneously hypertensive rats (SHRs) with an ACE2 inhibitor, MLN-4760 did not lead to decreased ACE2 activity, it triggered detrimental effects with respect to adiposity, the function of small arteries and angiogenesis, which were counterbalanced by the activation of compensatory mechanisms, including the Mas receptor, NO and H_2_S signaling stimulation.

The observed significant effect of MLN-4760 on the increase in body weight is in line with the previously published data of Bruce et al. [39], who found that activation of ACE2 via diminazene aceturate resulted in reduced adiposity and body weight reduction. The authors showed a decrease in the % of fat mass after ACE2 activation. To the best of our knowledge, this study is the first to show a direct pro-obesogenic effect of MLN-4760. Our finding is strengthened by significant negative correlations between body weight and visceral fat mass and alternative RAS and Ang 1–7 plasma concentrations in the MLN-4760 group. The absence of this correlation in the MLN-untreated group, as well as the absence of a difference in Ang 1–7 plasma concentrations between the SHR- and MLN-4760-treated groups, makes us speculate about alternative non-ACE2 routes of Ang 1–7 formation. It is likely that MLN-4760-induced inhibition of ACE2 activates neprilysin and/or thimetoligopeptidase-mediated Ang 1–7 formation. Alternatively, it might cause inhibition of Ang 1–7 degradation [23]. Apart from the above, our results suggest an important role of Ang 1–7 in the regulation of fat tissue metabolism in nonobese hypertensive rats. The decrease in visceral fat mass by increasing Ang 1–7 plasma concentrations is in accordance with data from the literature [40], and the results of our study suggest the deficiency of a favorable effect of Ang 1–7 on body weight and visceral adipose tissue mass (and circulating glucose) under conditions when tissue ACE2 function is compromised (e.g., SARS-CoV-2 infection). Moreover, the (pro)obesogenic effect of MLN-4760 could participate in the impairment of vascular function since increased adiposity and visceral obesity might be associated with hyperfunction of the arterial sympathetic nervous system [41].

As a next step, we evaluated the effect of MLN-4760 on vasoactive responses. The integrated blood pressure responses reflect the interplay between cardiac output and total peripheral vascular resistance. In this study, we found that the hypotensive response to acetylcholine and the participation of NO in blood pressure control remained unchanged after treatment with MLN-4760. Unlike in conduit arteries, where the acetylcholine-induced response is considered a marker of NO-mediated endothelial function, other vasodilator mediators, such as endothelium-derived hyperpolarizing factor or prostacyclin, could be dominant in smaller arteries and resistant vascular beds [42]. In our study, we confirmed that acute NO synthase inhibition led to a more pronounced decline in acetylcholine-induced BP, which agrees with findings that decreased NO production might be partially or fully substituted by a hyperpolarizing response to acetylcholine [43,44]. However, dysfunction of sympathetic nervous system activation was found in Wistar rats pretreated with L-NAME, which may also participate in the Ach-induced BP decrease in rats with reduced NO bioavailability [45]. Since treatment with MLN-4760 did not modify the hypotensive response, we suppose that the efficiency of NO-independent vasodilator pathways is not affected by MLN-4760, at least in vivo. On the other hand, the hypotensive response induced by the ACE inhibitor captopril was smaller in MLN-4760-treated rats, which could suggest the contribution of ACE2-mediated pathways to the antihypertensive effect of captopril mediated by Ang 1–7 (created by neutral endopeptidase neprilysin and/or thimetoligopepti-dase) via the Mas receptor. Considering that Ang 1–7, acting through Mas receptors, activates tissue kallikrein-kinins release and provides cardiovascular protection via bradykinin receptor-mediated effects [46], we suggest that MLN-4760 treatment could lead to the impairment of this protective action and reduce the hypotensive effect of captopril. The interaction between RAS and H_2_S signaling also could be taken into consideration. ACE is a metalloprotein, and Laggner et al. [46] proved that H_2_S can directly inhibit ACE activity by interfering with the zinc atom in the active center of ACE. In our previous study, we confirmed that captopril infusion diminished the effect of H_2_S on rat blood pressure, suggesting that ACE could be the common target of captopril and H_2_S action [47]. In this study, we confirmed the increased concentration of H_2_S in plasma and within the cardio-vascular system (heart) after treatment with MLN-4760, which can lead to the mild vasoconstrictor action of endogenous H_2_S observed in untreated rats switching to vasorelaxant action since the dual effect of H_2_S on vascular function was observed previously [48]. We suppose that stimulation of the sulfide signaling pathway could disable captopril action and partially mask its hypotensive effects.

Several studies confirmed that most arteries in SHRs had increased sympathetic innervation and elevated α1-adrenergic receptor-mediated contractile responses compared to normotensive rats [49]. The thoracic aorta, as a type of elastic artery, undergoes specific development during hypertension, and the hypertrophied arterial wall displays decreased contractile efficiency as a result of structural remodeling along with increased sensitivity of adrenergic receptors [11]. Although after treatment with MLN-4760, we did not find changes in vasoactive responses induced by noradrenaline in vivo, the increased maximal contraction and/or (in thoracic aorta) increased sensitivity to noradrenaline were confirmed in all isolated arteries in this study. Similarly, Lemos et al. [50] observed that endothelium-dependent contraction to phenylephrine was increased after blockade of Ang 1–7 in the aorta of mRen-2 rats, a monogenic model of hypertension attributed to elevated renin activity. The authors declared that the local accumulation of Ang II in arterial tissue of mRen-2 rats could contribute to the increased formation of Ang 1–7 and its vasoactive effects. Similar importance of local RAS also has been declared by Li et al. [51], who confirmed increased gene expression of all RAS components in the aorta of SHRs. The authors suggested that the tissue rather than circulating RAS contributes to hypertensive cardiovascular remodeling, whereby an increased ACE2 gene expression in cardiovascular tissues of SHR seemed to be a compensatory reaction. From this point of view and with respect to elevated systemic resistance of SHRs, the increased contractility and sensitivity of adrenergic receptors in isolated arteries after treatment with MLN-4760 could be considered a deleterious effect. In addition, the integrity of the vascular endothelium plays a significant role in vascular tone and blood pressure regulation. We and others previously confirmed that the prevalence of cyclooxygenase vasoconstrictors, overproduction of superoxide, and/or reduced levels of NO and hyperpolarizing factors participated in endothelial dysfunction, which was manifested as an elevated contractile response at high concentrations of muscarinic receptor agonists [10,11,48,52,53] (Figure 4d,g). In this study, we found different effects of MLN-4760 in arteries depending on their diameter (thoracic aorta > femoral artery > mesenteric artery). In the mesenteric artery, Ach-induced relaxation was significantly reduced after MLN-4760 treatment, suggesting endothelial dysfunction that could result in elevated peripheral arterial resistance. On the other hand, the relaxant responses of the femoral artery and thoracic aorta remained unchanged after MLN-4760, similar to the final BP, despite elevated contractile responses found in all arteries investigated in this study. Thus, other BP regulatory systems had to be affected, which helped to maintain BP at unaltered levels. One of the compensatory mechanisms could be associated with elevated Mas receptor protein expression in conditions of reduced ACE2 protein expression, which was associated with the inhibitory effect of Mas receptor signaling on contractile responses. Silva et al. [54] demonstrated that an exercise-induced increase in Mas receptor expression in SHR aortas improved Ang 1–7-mediated vascular function, acting as a counter regulator of classic Ang II/AT1-mediated effects. In untreated SHRs, Zhang et al. [55] showed that Mas receptor protein expression is decreased in arterial tissue and that stimulation of the Ang 1–7/Mas receptor axis by Ang 1–7 not only improved endothelium-dependent vasorelaxation but also inhibited contractile responses, which was associated with stimulation of the NO/cGMP signaling pathway. Our results also confirmed the increased participation of basally released NO in the suppression of contractile responses in the aortas of rats treated with MLN-4760. This was associated with a strong tendency of increased NOS activity as a result of the balance among unchanged eNOS, decreased nNOS and increased iNOS expression, although the alternative NOS-independent production of NO from nitrites and nitrates also cannot be ruled out [56]. Zheng et al. [57] confirmed in neuronal cell lines that an increase in ACE-2 expression caused an increase in nNOS expression, which agrees with our results of parallelly decreased expression of both proteins. On the other hand, although iNOS is typically expressed in response to cellular stress, Cheng et al. [58] suggested that upregulation of iNOS observed in aortas of SHRs could be a compensatory mechanism for the elevation of BP during the development of hypertension. Taken together, NO signaling was stimulated or at least fully functional and not disturbed after treatment with MNL-4760.

The other compensation seems to be the accentuation of H_2_S-mediated mechanisms since we found elevated levels of H_2_S in plasma and the heart. The decreased expression of H_2_S-producing enzymes in the MNL-4760 group could be explained by negative feed-back regulation to maintain a constant H_2_S level. Similar negative correlations between NOS expression and activity also have been confirmed by other authors [59,60,61]. Nevertheless, we confirmed that, unlike in control rats, H_2_S significantly participated in maintaining endothelial function in the MLN-4760 group (unlike in the control group). Similar compensatory crosstalk between NO and H_2_S signaling has already been declared in fructose-fed normotensive rats [62] as well as in SHRs, where the increased participation of H_2_S in vasorelaxation after acute NO inhibition could be considered a salvage mechanism in cases of endogenous NO deficiency [10]. Similarly, we confirmed a stronger anticontractile action of H_2_S produced by perivascular adipose tissue surrounding the thoracic aorta in hypertriglyceridemic rats [63], suggesting that H_2_S signaling can be stimulated as a balancing pathway in different pathological conditions. In addition, the influence of MLN-4760 on the autonomic nervous system was not investigated in this study. Thus, central compensatory mechanisms also may significantly contribute to the maintenance of BP at unaltered levels.

Furthermore, we investigated whether MLN-4760 may alter angiogenesis. We used the chick CAM model, a commonly used model for the in vivo study of both angiogenesis and antiangiogenesis in response to various factors. We found reduced vascular growth in MLN-4760-treated CAMs. Previously, it has been shown that reduced NO levels are associated with anti-angiogenic effects [21,22], and ACE2 inhibition is associated with reduced NO production in Mas receptor downstream pathways. Thus, our findings agree with the findings of impaired endothelial function found in the mesenteric arteries and suggest caution in the use of MLN-4760, as it may deteriorate the function of small arteries and angiogenesis in hypertensive sub-jects.

As SARS-CoV-2 enters the cells by binding to ACE2, one could assume that inhibition of ACE2 by suitable ACE2 inhibitor could block SARS-CoV-2 entry to the cells. On the other hand, simulation studies of Nami et al. [64] showed that the Spike protein could bind to ACE2 inhibited by MNL-4760 even with higher affinity than to the native ACE2. Nevertheless, Angeli et al. [65] commented that “blockade of ACE2 may be a clever way to eradicate the disadvantageous contribution of ACE2 as a viral entry route. “Our results suggest that short-term low-dose administration of ACE2 inhibitor could provide the way of SARS-CoV-2 entry reduction into the cells without overall detrimental consequences to organism.

## 5. Limitations

The main limitation of this study seems to be associated with the choice of ACE2 inhibitor dose. Selecting an appropriate dose of ACE2 inhibitor to reflect the consequences of inhibiting ACE2 receptors after they have been occupied by the virus is complicated. Further studies and comparisons of the effect of multiple doses of MLN-4760 on the cardiovascular system would probably be able to elucidate whether higher doses of inhibitor, longer duration of treatment, or different ACE2 inhibitors also will induce similar compensatory mechanisms, or if detrimental effects also will be manifested in the larger arteries, heart and/or brain.

## 6. Conclusions

In conclusion, our study revealed the complex action of a low dose of MLN-4760 in SHRs. On the one hand, MLN-4760 revealed a pro-obesogenic effect and was detrimental to small artery function and angiogenesis. On the other hand, compensatory mechanisms can be induced in larger arteries (and possibly also in other tissues), which were associated with Mas receptor stimulation as well as accentuation of NO and H_2_S signaling. Altogether, we showed that chronic low-dose MLN-4760-treatment failed to reduce ACE2 activity and did not alter BP while significant NO and H_2_S-mediated beneficial cardiovascular mechanisms were induced in conditions used in this study.

## Figures and Tables

**Table 1:** General characteristics of experimental animals.

Parameter	SHR	*n*	SHR + MLN	*n*
basal BW (g)	292.00 ± 3.69	20	296.20 ± 3.84	20
end BW (g)	309.65 ± 4.09	20	321.05 ± 4.42	20
Δ BW (%)	6.05 ± 0.44	20	8.38 ± 0.45 ***	20
basal SBP (mmHg)	160.78 ± 5.76	18	162.61 ± 5.53	18
end SBP (mm/hg)	170.06 ± 6.58	18	174.17 ± 7.21	18
Δ SBP (%)	5.87 ± 2.31	18	7.16 ± 2.83	18
HW (mg)	1148.42 ± 0.04	11	1209.49 ± 0.03	12
HW/BW (mg/g)	3.72 ± 0.14	11	3.78 ± 0.10	12
VF (mg)	7833.22 ± 331.9	12	8985.02 ± 300.45 *	12
VF/BW (mg/g)	25.1 ± 0.86	12	28.1 ± 0.92 *	12
ACE2 activity (mU/mg)	14.61 ± 3.54	12	19.30 ± 1.39	12
H_2_S plasma (µM)	11.9 ± 0.7	12	22.7 ± 2.4 ***	12
H_2_S heart (nmol/mg of protein) GLU (mmol/L)	1062 ± 61 6.97 ± 0.05	12 8	1534 ± 91 *** 7.13 ± 0.24	12 8
TG (mmol/L)	1.66 ± 0.09	8	1.70 ± 0.13	8
CHOL (mmol/(L)	2.10 ± 0.05	8	2.21 ± 0.06	8
HDL-C (mmol/L)	1.34 ± 0.04	8	1.41 ± 0.03	8

Abbreviations: BW, body weight; Δ BW, body weight gain; SBP, systolic blood pressure; Δ SBP, the increment of systolic blood pressure; HW, heart weight; HW/BW, heart weight/body weight ratio; VF, visceral fat weight; VF/BW, visceral fat weight/body weight ratio; GLU, glucose; TG, triacylglycerides; CHOL, total cholesterol; HDL-C, high-density lipoprotein cholesterol. Values are the mean ± S.E.M.; * *p* < 0.05 and *** *p* < 0.001 vs. SHRs; differences between groups were analyzed by Student’s *t*-test.

**Table 2:** The equilibrium angiotensin peptides levels in plasma.

Parameter	SHR	*n*	SHR + MLN	*n*
Ang I (1–10) (pmol/L)	94.53 ± 6.44	7	105.93 ± 9.67	7
Ang II (1–8) (pmol/L)	161.05 ± 11.94	7	186.42 ± 13.13	7
Ang 1–7 (pmol/L)	6.61 ± 0.98	7	6.36 ± 0.89	7
Ang 1–5 (pmol/L)	20.27 ± 1.25	7	20.89 ± 2.09	7
Ang IV (3–8) (pmol/L)	8.09 ± 0.73	7	10.16 ± 0.85	7

Angiotensin product/substrate ratios for a certain enzymatic cleavage directly reflect the state of soluble RAS activity. Abbreviations: Ang I, angiotensin 1; Ang II, angiotensin II; Ang 1–7, angiotensin 1–7; Ang 1–5, angiotensin 1–5; Ang IV, angiotensin IV.

## Data Availability

The data presented in this study are available on request from the corresponding author.

## Supplementary Materials

The following are available online at https://www.mdpi.com/article/10.3390/biomedicines10010038/s1, Supplementary File S1: Preparation of ex ovo chick CAM.
