# Supplementary material for: Vascular Effects of Low-Dose ACE2 Inhibitor MLN-4760—Benefit or Detriment in Essential Hypertension?
Source: Biomedicines. 2021 Dec 24;10(1):38. doi: 10.3390/biomedicines10010038 (PMC8773407; doi:10.3390/biomedicines10010038)
Supplement: Supplementary file 1 [file biomedicines-10-00038-s001.zip › biomedicines-1516668-supplementary.pdf]

Supplementary File S1.

Vascular effects of low dose ACE2 inhibitor MLN-4760 – Benefit or detriment in essential hypertension?

Andrea Berenyiova[1], Iveta Bernatova[1], Anna Zemancikova[1], Magdalena Drobna[1], Martina Cebova[1], Samuel Golas[1], Peter Balis[1], Silvia Liskova[1,3], Zuzana Valaskova[1,4], Katarina Krskova[2], Stefan Zorad[2], Ezgi Dayar[1], and Sona Cacanyiova[1,*]

Preparation of ex ovo chick chorioallantoic membrane (CAM)

1. Ex ovo model of CAM

Fertilized Leghorn chicken white eggs were purchased from a local producer (Liaharenský podnik a.s., Nitra, Slovakia). The eggs were cleaned with sterile water and kept at 15 °C for 4 days. The cultivation of eggs started on the fifth day after laying, when the eggs were removed from the incubator, kept at room temperature (approximately 25 °C) for 4 h and then for 2 h at 29–30 °C in an incubator (myTemp™, Benchmark Scientific, Edison, New Jersey, USA). Then, the eggs were placed in a vertical position, with the round tip up, in an automatic egg incubator at 37.8 °C, 65–70% humidity and rotation (35° angle of rotation every 120 min) for 4 days.

After 96 h of incubation (on the 5th day of embryonal development, D5), the eggs were removed from the automatic incubator and placed in an incubator (Memmert, Schwabach, Germany) for 2 h in a horizontal position without rotation at a temperature of 37.8 °C and a humidity of 60–65%. The eggs were then candled, and the yolk line was drawn with a pencil on the shell. Then, the eggs were held in this position until cracking. Subsequently, the eggs were placed in a laminar box (Helago, A&D, Japan), the bottom side of the shell was cleaned with 75% ethanol, the shell was gently sawn with a laboratory wheel saw (avoiding shell cracks in the area of yolk), and the egg content was gently placed in a sterilized polypropylene medical cup. After placement of the egg content in the cup, $CaCO_3$ was added (10 mg, outside the yolk area), and the cups were covered with food foil using a sterilized rubber band. Approximately 30–35 holes were made in the foil using a sterile 18G needle, and the cups were placed in the Memmert IF160 incubator at 37.5 °C and 60–65% humidity for 72 h. During incubation, embryos were visually examined every day, and dead embryos were excluded daily from the experiment.

2. Drug application

On D8, polytetrafluoroethylene (Teflon) circles were placed on the surface of developing CAM for 72 hours. On the underside of the circles, 0.45% methylcellulose (MC) gel containing 10% DMSO (control) or 5 μg MLN-4760 diluted in 10% DMSO was applied (n = 16 in each group).

After 72 h of incubation, CAM status was examined with a Motic SMZ171 trinocular stereo microscope (Motic, Kowloon, Hong Kong) using a Bresser MikroCamII 12 MP, USB 3.0, microscope camera (Bresser, Rhede, Germany) and 0.65 c-mount. Areas of the circles were photographed before and after removal of the Teflon circle.

The pictures were then evaluated using MicroCamLabII (Bresser, Rhede, Germany) software. To improve the image, the native colors were inverted by software to blue color, and the contrast was

increased to achieve the best possible resolution. All vascular branching points were counted in the area of contact with the applied substance (A_{Appl}, ~6.2 mm diameter) and in the area adjacent to A_{Appl} (A_{Adj}, diameter ~9.3 mm, i.e., plus 50% of A_{Appl} diameter), and the number of branchings/mm^2 was counted in each area. Each CAM was examined by two evaluators, and the average values of their evaluations were statistically evaluated (n = 8 per group). At the end of the experiment, the embryos were killed by decapitation. The preliminary death of embryos was similar in both groups (50%).

3. Preparation of Teflon circles

The circles were cut out with a paper punch from a commercially available food grade Teflon foil. The diameter of the circle was ~6.2 mm. The exact diameter and area were measured using MicroCamLabII (Bresser, Rhede, Germany) software. The rings were sterilized in 75% ethyl alcohol overnight, dried in a laminar box and sterilized by UV-C light in a laminar box for at least 10 minutes on each side. Ten microliters of MC gel (control or containing MLN-4760) was placed on the ring, and the gel was dried in a laminar box until completely dry (~150 min).

The advantage of Teflon circles over paper circles or MC discs, alone or with nylon mesh, is that the edge of the disc is clearly visible, the disc does not sink into the contents of the egg, it does not move spontaneously on the surface of the CAM during CAM growth, it can be relatively easily removed from the CAM surface at the end of the experiment, and vascularization can be easily investigated. This allows the examination of the growth of the blood vessels under the surface of the disc, which were in direct contact with the test drug, in comparison with the adjacent and/or far areas of the CAM.

4. Preparation of medical cups

Before use, the white polypropylene medical cups (80 mL, 74 mm width, 30 mm depth) were washed with 75% ethanol, dried in a laminar box and, after drying, exposed to UV-C light for 4 x 30 min (two times from the inside and two times from the outside) in a laminar box.
